# Exploring the mechanisms of action of human secretory RNase 3 and RNase 7 against *Candida albicans*

**DOI:** 10.1002/mbo3.373

**Published:** 2016-06-08

**Authors:** Vivian A. Salazar, Javier Arranz‐Trullén, Susanna Navarro, Jose A. Blanco, Daniel Sánchez, Mohammed Moussaoui, Ester Boix

**Affiliations:** ^1^ Department of Biochemistry and Molecular BiologyFaculty of BiosciencesUniversitat Autònoma de BarcelonaCerdanyola del VallèsE‐08193Spain; ^2^ Institut de Biotecnologia i BiomedicinaUniversitat Autònoma de BarcelonaCerdanyola del VallèsE‐08193Spain

**Keywords:** Cytotoxicity, host–pathogen interactions, infectious diseases, innate immunity

## Abstract

Human antimicrobial RNases, which belong to the vertebrate RNase A superfamily and are secreted upon infection, display a wide spectrum of antipathogen activities. In this work, we examined the antifungal activity of the eosinophil RNase 3 and the skin‐derived RNase 7, two proteins expressed by innate cell types that are directly involved in the host defense against fungal infection. *Candida albicans* has been selected as a suitable working model for testing RNase activities toward a eukaryotic pathogen. We explored the distinct levels of action of both RNases on yeast by combining cell viability and membrane model assays together with protein labeling and confocal microscopy. Site‐directed mutagenesis was applied to ablate either the protein active site or the key anchoring region for cell binding. This is the first integrated study that highlights the RNases’ dual mechanism of action. Along with an overall membrane‐destabilization process, the RNases could internalize and target cellular RNA. The data support the contribution of the enzymatic activity for the antipathogen action of both antimicrobial proteins, which can be envisaged as suitable templates for the development of novel antifungal drugs. We suggest that both human RNases work as multitasking antimicrobial proteins that provide a first line immune barrier.

## Introduction

Fungal infections are a threat to hospitalized and immunocompromised patients. *Candida albicans* is a major common fungal pathogen in humans that colonizes the skin and the mucosal surfaces of most healthy individuals. Together with superficial infections, such as oral or vaginal candidiasis, a life‐threatening systemic infection can eventually occur (Mayer et al. 2013). *Candida albicans* is the causative agent of most candidiasis, but other emerging species, such as *Candida glabrata* and *Candida krusei*, are also considered to be threats to patient populations. *Candida* infections have increased dramatically over the last two decades. Considering the increase in *Candida* pathogenesis, mostly in immunocompromised patients, but also in healthy individuals, active research has focused on new therapies and treatments. Several factors and activities that contribute to the pathogenic potential of this fungus have been identified. As a first consideration, *Candida albicans* displays a complex cell wall organization that plays a role in maintaining structural integrity and mediating adherence. Its specific composition, which is predominantly composed of carbohydrates (Chitin, *β*‐1,3 glucan, and *β*‐1,6 glucan), offers resistance to host molecular defense and is impermeable to most potential antifungal drugs (Molero et al. 1998; Mayer et al. 2013).

Knowledge of pathogenicity mechanisms, ranging from cell wall complexity to the adhesion and host cell invasion mechanism (Chaffin 2008), is crucial for the rational design of novel antifungal drugs (Molero et al. 1998; Mayer et al. 2013). Antimicrobial peptides (AMPs) and in particular, those secreted by the human skin, our first natural barrier against infections, are regarded as appealing candidates for applied antifungal therapy (den Hertog et al. 2005; Vylkova et al. 2007; Andrès 2012). Indeed, AMPs offer a chemical defense system that protects the skin from potential pathogenic microorganisms threatening to colonize host tissues (Harder and Schroder 2002; Bardan et al. 2004; Gläser et al. 2005). Among skin AMPs, there are peptides with reported antifungal activity, such as cathelicidins (López‐García et al. 2005) and defensins (De Smet and Contreras 2005). Both peptides are rapidly released at high local concentrations when needed in response to infection or epidermal injury (Dorschner et al. 2001; Niyonsaba and Ogawa 2005; Sørensen et al. 2006). Moreover, the constant level of some constitutively produced antimicrobial peptides and proteins at skin surfaces suggests that these AMPs have been optimized during evolution to protect the skin surface from infection (Schröder and Harder 2006). In particular, human RNase 7 is one of the main products that is constitutively released by keratinocytes (Schröder and Harder 2006). RNase 7 not only has a well‐documented bactericidal activity (Harder and Schroder 2002; Torrent et al. 2010a; Pulido et al. 2013b) but also inhibits the growth of yeast (Harder and Schroder 2002; Huang et al. 2007) and dermatophytes (Fritz et al. 2012).

**Figure 1:**
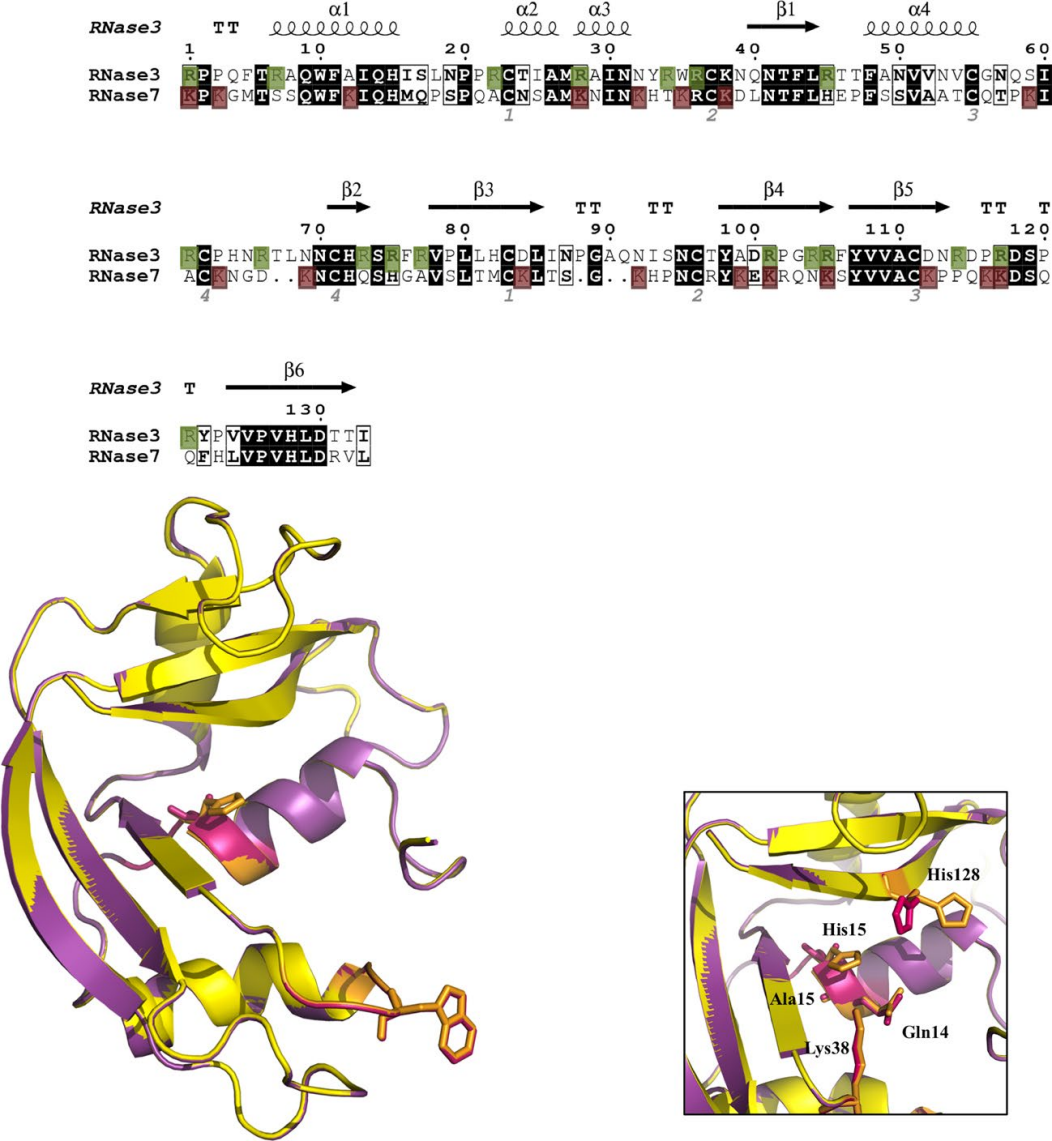
(A) Sequence alignment of RNase 3 and RNase 7. Primary sequences (UniProt codes: P12724 and Q9H1E1) were used, respectively. RNase 3 three‐dimensional structure is indicated (PDB ID: 4OXF). Cationic residues are shown in both proteins in green and fuchsia boxes, respectively. The alignment was performed using the ESPript program (http://espript.ibcp.fr/EsPript/). (B) Three dimensional representation of crystal structures of wild‐type RNase 3 (yellow; PDB ID: 4OXF) and active site mutant RNase 3‐H15A (purple; PDB ID: 4OWZ). Mutated residues (His 15 and Trp 35) are depicted in baton sticks. (C) Detail of active centre in both proteins. Picture was drawn with *PyMOL* Molecular Graphics System (Schrödinger, LLC).

Interestingly, RNase 7 is a member of the RNase A superfamily (Fig. 1). This family, which includes other secretory RNases with antimicrobial properties (Boix and Nogués 2007), is a protein family that is suggested to have emerged with an ancestral host defense role (Pizzo and D'Alessio 2007; Rosenberg 2008). Antimicrobial RNases are expressed by epithelial tissues and blood cell types, and their expression can be induced by inflammatory agents and bacterial infection (Gupta et al. 2012; Spencer et al. 2013; Amatngalim et al. 2015; Becknell et al. 2015). In particular, RNase 3 and RNase 7 are the main representative members that show a high bactericidal activity (Torrent et al. 2010a, 2012; Pulido et al. 2013b). RNase 7 is expressed in the skin‐derived stratum, the gut, and the respiratory and genitourinary tracts (Spencer et al. 2013; Becknell et al. 2015) and is particularly active against both gram‐negative and gram‐positive species, such as *Enterococcus faecium, Pseudomonas aeruginosa,* and *Escherichia coli* (Harder and Schroder 2002; Huang et al. 2007; Torrent et al. 2010b).

On its turn, RNase 3, another of the main antimicrobial RNases within the RNase A superfamily (Fig. 1), also called the Eosinophil Cationic Protein (ECP), is involved in inflammatory processes mediated by eosinophils and is released by the secondary granules upon infection (Acharya and Ackerman 2014). RNase 3 has also been reported to display high antimicrobial activity against both gram‐negative bacteria, such as *E. coli, Acinetobacter baumannii,* and *Pseudomonas sp*. (Torrent et al. 2011b), and gram‐positive species, such as *Staphylococcus aureus, Micrococcus luteus,* and *E. faecium*, (Torrent et al. 2011b) as well as *Mycobacteria* (Pulido et al. 2013b).

In this work, we explored the antifungal properties of both RNase 3 and 7. We used *C. albicans* as a eukaryotic pathogen model, which has proven to be an appropriate first approach to understand the distinct levels of action of antimicrobial RNases.

## Experimental Procedures

### Protein expression and purification

Recombinant RNase 3 and RNase 7 were expressed in *E. coli* BL21 (DE3) using the pET11c plasmid vector as previously described (Torrent et al. 2009). Protein expression, solubilization from inclusion bodies, refolding, and purification steps were carried out as described (Boix et al. 1999). RNase 3‐H15A, RNase 3‐W35A, and RNase 7‐H15A variants were constructed using the Quick Change Site‐Directed Mutagenesis kit (Stratagene, La Jolla, CA). All constructs were confirmed by DNA sequencing and the purified protein was analyzed by MALDI‐TOF MS and *N*‐terminal sequencing.

### Crystal structure determination

Recombinant protein was purified by cation exchange and reverse phase chromatographies as previously described (Boix et al. 2012a). RNase 3‐H15A mutant was crystallized following the conditions for wild‐type RNase 3 modified from (Mallorquí‐Fernández et al. 2000). Protein sample was lyophilized and resuspended at 12 mg/mL in 20 mmol/L sodium cacodylate pH 5.0 and equilibrated against 7% Jeffamine^™^ M‐600, 0.1 mol/L Na citrate, pH 5.2, 10 mmol/L FeCl_3_. One microliter of the sample was mixed with an equal volume of the reservoir solution and set to incubation at 20°C. After 5 to 10 days, crystals appeared and were soaked using 30% methyl pentanediol as cryofreezing agent. Data were captured at 100 K using a *λ*_XRD_ = 0.9795 Å at the BL13 beamline of the ALBA Synchrotron Light Facility (Spain). XDS (Kabsch, 2010) was used for data processing. Scaling was performed with SCALA and molecular replacement with PHASER (McCoy et al., 2007) using as a model the RNase 7 NMR structure (PDB coordinate file 2HKY (Huang et al. 2007)). Iterative cycles of refinement and manual structure fitting were performed with PHENIX (Adams et al., 2010) and COOT (Emsley and Cowtan, 2004 ) until *R*_*free*_ could not be further improved (Brunger, 1992 ). Finally, the stereochemistry of the structure was validated with SFCHECK (Vaguine et al., 1999 ). Table S1 shows all the data collection and structure refinement statistics. Solvent accessible surface areas for protein residues were calculated using the *Areaimol* software (The CCP4 suite: programs for protein crystallography, 1994).

### Enzymatic activity analysis

The RNases enzymatic activities of RNase 3, RNase 7, and active site mutants were measured spectrophotometrically by registering the degradation of the oligocytidylic acid (Cp)_4_C>p at 286 nm in a Cary Eclipse spectrophotometer. The (Cp)_4_C>p acid, purified from poly(C), was used as a substrate, as previously described (Boix et al. 1999). Assay conditions were 1 *μ*mol/L protein, 84 *μ*mol/L oligocytidylic acid in 0.2 mol/L NaAcO, pH 5.0, 3 min incubation time, and 25°C. Alternatively, a zymogram analysis was performed using a 15% SDS‐PAGE gel containing polyuridylic acid, poly(U), as a substrate according to the method previously described (Bravo et al. 1994).

### Model membrane leakage activity

Membrane leakage activity was assessed by ANTS/DPX (8‐aminonaphthalene‐1,3,6‐trisulfonic acid disodium salt/p‐xylenebispyridinium bromide) as previously (Torrent et al. 2007). Large unilamellar vesicles of dioleoyl‐phosphatidyl choline: dioleoyl‐phosphatidyl glycerol (3:2 molar ratio), containing 12.5 mmol/L ANTS and 45 mmol/L DPX in 20 mmol/L NaCl, 10 mmol/L Tris/HCl, pH 7.5, were diluted to 30 *μ*mol/L and incubated at 25°C with the proteins for 45 min. Leakage was monitored as the increase in fluorescence (*λ*_excitation_ = 386 nm; *λ*_emission_ = 535 nm).

### Fluorescent labeling of RNases

RNase 3, RNase 3‐H15A, RNase 3‐W35A, RNase 7‐H15A, and RNase 7 were labeled with Alexa Fluor 488 Labeling kit (Molecular Probes, Invitrogen, Carlsbad, CA), following the manufacturer′s instruction as previously described (Torrent et al. 2010a). To 0.5 mL of 2 mg/mL protein solution in phosphate saline buffer (PBS), 50 *μ*L of 1 mol/L sodium bicarbonate, pH 8.3, was added. The protein was incubated for 1 h at room temperature with the reactive dye, with stirring, and the labeled protein was separated from the free dye by PD‐10 desalting column.

### Candida albicans growth conditions

*Candida albicans* (ATCC 90028) cells were maintained at −70°C and incubated overnight with agitation at 37°C in Sabouraud Dextrose broth (mycological peptone, glucose, pH 5.6) Fluka‐Sigma S3306. Previous to each assay, cells were subcultured for ~2–3 h to yield a midlogarithmic culture.

### Minimum fungicidal concentration

*Candida albicans* ATCC 90028 was cultured overnight in Sabouraud Dextrose broth at 37°C and subcultured the next day in fresh Sabouraud and grow to an optical density of 0.4 at 600 nm (mid log‐phase). Cells were washed twice in nutrient broth or PBS, and diluted to ~2 × 10^6^ cells/mL. Proteins serially diluted were added to 2 × 10^5^ cells from 20 to 0.1 *μ*mol/L final concentration. *Candida albicans* was incubated at 37°C during 4 h in Sabouraud nutrient broth, PBS or 10 mmol/L sodium phosphate buffer, pH 7.5. Following, the samples were plated onto Sabouraud Petri dishes and incubated at 37°C overnight. Antifungal activity was expressed as the MFC, defined as the lowest protein concentration required for more than 99% of microorganism killing. MFC of each protein was determined from two independent experiments performed in triplicate for each concentration.

### Cell viability assay

Antimicrobial activity was also assayed by following the cell viability of *C. albicans*, using the BacTiter‐Glo™ Microbial Cell Viability kit (Promega, Madison, WI), which measures the number of viable fungal cells, by ATP quantification. ATP, as an indicator of metabolically active cells, is indirectly measured by a coupled luminescence detection assay. The luminescent signal is proportional to the amount of ATP required for the conversion of luciferin into oxyluciferin in the presence of luciferase.

An overnight culture of *C. albicans* was used to inoculate fresh Sabouraud liquid culture, and logarithmic phase culture was grown to an OD_600_ of 0.2. RNase 3, RNase 7, and mutants were added to 0.1 mL of cell culture at a final concentration from 0.025 to 20 *μ*mol/L. The *C. albicans* viability was followed after 4 h of incubation at 37°C. 50 *μ*L of incubation culture was mixed with 50 *μ*L of BacTiter‐Glo™ reagent in a microtiter plate following the manufacturer instructions and incubated at room temperature for 10 min. Luminescence was read on a Victor3 plate reader (PerkinElmer, Waltham, MA) with a 1‐s integration time. IC_50_ values were calculated by fitting the data to a dose–response curve.

### Cell survival assay

*Candida albicans* viability assay was performed using the Live⁄Dead^®^ microbial viability kit as previously described (Torrent et al. 2010a). *Candida* strain was grown at 37°C to ~5 × 10^6^ cells/mL, centrifuged at 5000 × g for 5 min and resuspended in a 0.85% NaCl solution, in accordance with the manufacturer instructions. *Candida albicans* cell culture was stained using a SYTO^®^9/propidium iodide 1:1 mixture. SYTO^®^9 is a DNA green fluorescent dye that diffuses thorough intact cell membranes and propidium iodide is a DNA red fluorescent dye that can only access the nucleic acids of membrane damaged cells, displacing the DNA bound SYTO^®^9. The method allows the labeling of intact viable cells and membrane compromised cells, which are labeled in green and red, respectively, referred to as live and dead cells. The viability kinetics was monitored using a Cary Eclipse Spectrofluorimeter (Varian Inc., Palo Alto, CA). Cell viability profiles were registered after adding from 1 to 5 *μ*mol/L of final protein concentration. To calculate the cell viability, the fluorescence in the range of 510–540 nm and 620–650 nm were integrated to obtain the SYTO^®^9 (live cell) and the propidium iodide (dead cell) signals, respectively. Then, the percentage of live bacteria was represented as a function of time.

### Cell viability by confocal microscopy

The kinetics of *C. albicans* survival was followed by confocal microscopy for 180 min at 37°C. Experiments were carried out in a plate‐coverslide system. Two hundred and fifty microliters of *C. albicans* ~ 4 × 10^6^ cells/mL were stained as described below and mixed with the protein at 5 *μ*mol/L final concentration. *Candida albicans* cell cultures were prestained using the SYTO^®^9⁄propidium iodide 1:1 mixture provided in the Live ⁄Dead^®^ staining kit (Molecular Probes, Eugene, OR). Confocal images of the yeast culture were captured using a laser scanning confocal microscope (Leica TCS SP2 AOBS equipped with a HCX PL APO 63, ×1.4 oil immersion objective; Leica Microsystems, Wetzlar, Germany). SYTO^®^9 was excited using a 488 nm argon laser (515–540 nm emission collected) and propidium iodide was excited using a diode pumped solid‐state laser at 561 nm (588–715 nm emission collected). To record the time‐lapse experiment, Life Data Mode software (Leica) was used, obtaining an image every minute.

Alternatively, labeled protein distribution in cell cultures was followed by confocal microscopy. 300 *μ*L of *Candida albicans* yeast (~4 × 10^6^ cell/mL) was incubated with Alexa‐labeled proteins at 1 to 5 *μ*mol/L during 1 h in PBS. Previously, cells were washed with PBS and labeled with Hoescht 33342 at 0.5 *μ*g/mL for 10 min before observation of unfixed cells in Leica TCS SP5 AOBS equipped with a PL APO 63 × 1.4‐0.6 CS oil immersion objective (Leica Microsystems, Mannheim, Germany). Fluorochromes were excited by 405 nm (Hoechst 33342) and 488 nm (Alexa Fluor 488 nm) and both emissions collected with a HyD detector. Alexa Fluor 488‐labeled proteins were added directly to the cultures and time lapse was recorded at intervals of 30 sec for 1 h.

### Cell membrane depolarization assay

Membrane depolarization was assayed by monitoring the DiSC_3_(5) fluorescence intensity change in response to changes in transmembrane potential as described previously (Torrent et al. 2008). *Candida albicans* cells were grown at 37°C to the midexponential phase and resuspended in 5 mmol/L Hepes‐KOH, 20 mmol/L glucose and 100 mmol/L KCl at pH 7.2. DiSC3(5) was added to a final concentration of 0.4 *μ*mol/L. Changes in the fluorescence for alteration of the cell plasma membrane potential were continuously monitored at 20°C at an excitation wavelength of 620 nm and an emission wavelength of 670 nm. When the dye uptake was maximal, as indicated by a stable reduction in the fluorescence as a result of quenching of the accumulated dye in the membrane interior, protein in 5 mmol/L Hepes‐KOH buffer at pH 7.2 was added at a final protein concentration from 1 to 5 *μ*mol/L and incubated for 50 min. Maximum depolarization was calculated when the fluorescence signal was fully stabilized and the depolarization percentage was calculated taking Triton X‐100 at 10% as a positive control. The time required to reach a stabilized maximum fluorescence reading was recorded for each condition, and the time required to achieve half of total membrane depolarization was estimated from the nonlinear regression curve. All conditions were assayed in duplicate.

### Cell membrane permeabilization activity

Membrane permeabilization was evaluated by using the Sytox^*®*^ *Green* uptake assay. *Sytox* ^*®*^ *Green* is a cationic cyanine dye (≈900 Da) that is not membrane permeable. When a cell's plasma membrane integrity is compromised, influx of the dye, and subsequent binding to DNA, causes a large increase in the fluorescence signal. For *Sytox* ^*®*^ *Green* assays, *Candida albicans* cells were grown to midexponential growth phase at 37°C and then centrifuged, washed, and resuspended in PBS. Cell suspensions in PBS (~2 × 10^6^ cells/mL) were incubated with 1 *μ*mol/L *Sytox* ^*®*^ *Green* for 15 min in the dark prior to the influx assay. At 2 to 4 min after initiating data collection, proteins at 1 and 5 *μ*mol/L final concentration were added to the cell suspension, and the increase in *Sytox* ^*®*^ *Green* fluorescence was measured (excitation wavelength at 485 nm and emission at 520 nm) for 50 min in a Cary Eclipse spectrofluorimeter. Bacterial cell lysis with 10% Triton X‐100 was taken as the maximum fluorescence reference value.

### Cell binding assay

*Candida albicans* cells were cultured overnight in Sabouraud Dextrose broth at 37°C and subcultured the next day in fresh Sabouraud and grow to an optical density of 0.4 at 600 nm (mid log‐phase). Cells were washed twice in PBS, and diluted to ˜2 × 10^6^ cells/mL. Cells were incubated in 100 *μ*L of PBS at 37°C with proteins at 1 *μ*mol/L final concentration during different periods of time up to 1 h. Following, the samples were centrifugated at 13000 rpm. The supernatant samples were concentrated using 10 kDa cut‐off filters to 20 *μ*L. The presence of the proteins was checked by 15% SDS‐PAGE and Coomassie Blue staining. Reference protein controls were treated following the same protocol in the absence of cells.

### Fluorescent‐assisted cell sorting (FACS) assay

The evolution of cell population was followed by cell cytometry. *Candida albicans* cells were grown at 37°C to midexponential phase (~2 × 10^6^ cells/mL), centrifuged at 5000 × g for 2 min, resuspended in PBS. 500 *μ*L aliquot of the yeast suspension was incubated with 1 to 5 *μ*mol/L of Alexa‐Fluor‐labeled protein for 60 min. After incubation, 25,000 cells were washed three times with PBS buffer and subjected to FACS analysis using a FACSCalibur cytometer (BD Biosciences, Franklin Lakes, NJ) and excitation and emission wavelengths of 488 nm and 515–545 nm, respectively. Internal fluorescence uptake was evaluated at 2, 5, 15, and 60 min in 10,000 cells. Dead cells were stained with PI dye added at a final concentration of 10 *μ*g/mL.

### Cellular RNA degradation

A *C. albicans* cell culture suspension (~6 × 10^6^ cells/mL) was incubated in PBS and treated with the proteins at 3 *μ*mol/L final concentration for 30, 60, and 120 min. After incubation, cells were sedimented and resuspended in lysis buffer, 10% SDS and Phenol:Chloroform: isoamyl alcohol (IAA) and mixed with zirconia beads. RNA isolation was done using the Ribopure Yeast kit (Invitrogen) according to manufacturer's instructions. Samples were analyzed in a high sensitivity nucleic acid microfluidic chip using an *Experion* automated electrophoresis system (Bio‐Rad, Madrid, Spain). Cellular RNA populations were quantified by virtual gel densitometry.

### Statistical analysis

Results are reported as mean ± SD. Each mutant was compared with the corresponding wild‐type protein. Statistical analysis was performed by the paired Student's t‐test using STATA 11 software and IBM SPS 19 software. One‐way analysis of variance (ANOVA) was applied. *P* values <0.05 were considered significant.

## Results

### *Human RNases against* Candida albicans

The antifungal mechanisms of action of RNase 3 and RNase 7 on *Candida albicans* were characterized through a variety of methodological approaches. This is the first direct report of the involvement of both enzymatic and membrane damage activities in the RNases’ antimicrobial activity. Single‐point mutations that ablate either the protein catalytic (H15A) or cell‐binding (W35A) activities were designed to evaluate the distinct protein properties.

The protein toxicity on yeast cells was first analyzed by plotting the colony‐forming unit (CFU) reduction as a function of the protein concentration. Both RNases showed an effective protein concentration in the low micromolar range (Table 1), achieving minimal fungicidal concentrations (MFC_100_) at approximately 2 to 4 *μ*mol/L (30–60 *μ*g/mL), as evaluated in nutrient media (Sabouraud broth) and phosphate saline buffer (PBS) (Table 1). The antifungal activity was also assessed by a cell viability assay based on the quantification of the ATP levels that revealed similar IC_50_ values for both RNases. The calculated antifungal activity was comparable to the previously reported bactericidal activity (Torrent et al. 2012). Equivalent MFCs were achieved for low and high ionic strength phosphate buffers (Table S2), as previously observed for RNase 3 bactericidal activity (Torrent et al. 2012). However, the addition of increasing amounts of Ca^2+^ or NaCl to the nutrient growth media impaired considerably the RNases fungicidal activity (Table S2), as previously reported for the bactericidal action of RNase 3 (Lehrer et al. 1989) and other AMPs (Krishnakumari et al. 2009). Binding to *Candida* cells was assessed by monitoring the free unbound protein after incubation at sublethal concentrations in PBS for 1 h at 37°C (Fig. S1). No binding was achieved in equivalent assay conditions by the homologous RNase A, used here as a non antimicrobial reference protein.

**Table 1:** Antifungal activities of RNase 3, RNase 7, and mutant variants on *Candida albicans*

Protein	MFC_100_ (*μ*mol/L)		IC_50_ (*μ*mol/L)
	Sabouraud Broth	PBS	
RNase 7	2.5–5	2.5	1.60 ± 0.09
RNase 7‐H15A	3.5–5	2.5–5	1.93 ± 0.07*
RNase 3	2.5	2.5	2.50 ± 0.01
RNase 3‐H15A	5–10	5–10	3.45 ± 0.08**
RNase 3‐W35A	>20	>20	9.03 ± 0.52**

Minimal fungicidal concentration (MFC_100_) values were calculated by CFU counting on plated Petri dishes as described in the methodology. *C. albicans* cultures were treated with the proteins diluted in either the Sabouraud nutrient growth media or in a phosphate saline buffer (PBS). IC_50_, given as mean ± SD, were determined using the Bactiter‐Glo™ kit as detailed in the Experimental Procedures. Values are averaged from three replicates of two independent experiments. For the comparison of numerical variables between wild‐type and mutant, the Student's t‐test was used.

Values of *P *<* *0.05* and *P* < 0.009** are indicated.

**Figure 2:**
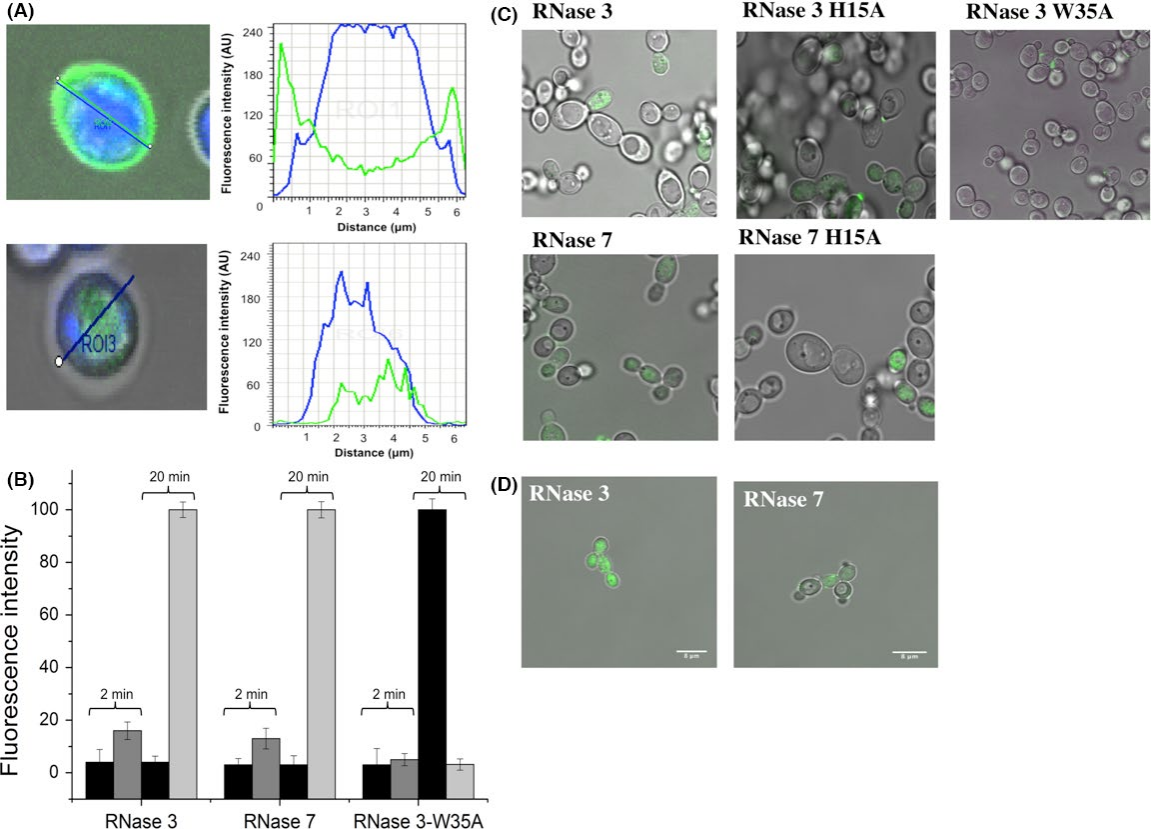
Confocal microscopy analysis of *Candida albicans* cell culture (~3 × 10^6^ cells/mL) incubated with 1 *μ*mol/L of RNase 3, RNase 7 and mutants labeled with Alexa Fluor 488 (green). (A and B) Cells were stained with Hoescht 33342 dye (blue) following the assay incubation conditions detailed in the Experimental Procedures section. Fluorescence and differential interference contrast (DIC) merge images were taken. Analysis was made at 2 and 20 min after protein addition at 1 *μ*mol/L final concentration. After protein addition, the evolution of the fluorescence signals was analyzed by confocal microscopy. A total of 20 cells were analyzed by regions of interest (ROIs) using the Leica TCS software. Yeast size mean was adjusted according to Hoescht‐labeled distribution and disc image. The cell mean size was around 4.5 *μ*m and a distance between 4.5 and 8 *μ*m was ascribed to the cell environment. (A) Profiles of fluorescence intensity for Alexa Fluor 488‐labeled protein (green) and *C. albicans* cells stained with Hoescht 33342 (blue). Examples of fluorescence profiles are shown at 2 min and 20 min for RNase 3. (B) Bar graphs of total internal and external fluorescence intensity values (maximum peak) are shown. Black bar corresponds to outer fluorescence and gray and light gray bar to inner fluorescence at 2 and 20 min, respectively. (C and D) Confocal microscopy analysis of *Candida* cell culture (~3 × 10^6^ cells/mL) incubated with 1 *μ*mol/L of RNase 3, RNase 7 and mutants labeled with alexa fluor 488. Distribution of Alexa Fluor 488‐labeled protein in treated *C. albicans* cells visualized by confocal microscopy, (C) Protein localization in yeast cells after 20 min of incubation at 37°C with labeled proteins. (D) Merged images after additional PBS washes to eliminate fluorescence background and free‐labeled proteins. The images were taken using a Leica TCS SP5 AOBS microscope.

Additionally, RNase 3 and RNase 7 action at the cell plasma membrane was evaluated by assessing their ability to trigger the depolarization and permeabilization of the cell membrane (Table 2). Complementarily, studies of both the kinetics of the depolarization and permeabilization activities of the enzymes were compared. Furthermore, a time course monitoring of the protein toxicity on the yeast cell population was completed using the Live/Dead^®^ staining kit, where a major reduction in the cell survival percentage was shown after 1 h (Fig. S3).Next, we labeled the recombinant proteins with an Alexa Fluor fluorescent marker and tracked the location of the proteins in cell cultures, in which the cell nuclei were stained with Hoechst stain (Fig. S2). The protein distribution was analyzed and it was observed that the protein and cell signals colocalized (Fig. 2). The protein internalization was also visualized at sublethal concentrations and short incubation times after removal of any remaining free protein (Fig. 2D).

**Table 2:** Cell membrane depolarization and permeabilization activities of RNases on *Candida albicans*

Protein	Max. membrane depolarization (AU)^1^	Membrane depolarization (%)^2^	Max. membrane permeabilization (AU)^1^	Membrane permeabilization (%)^2^
RNase 7	165.77 ± 1.10	71.67 ± 0.1	134.56 ± 1.95	45.90 ± 0.5
RNase 7‐H15A	153.96 ± 1.65*	66.54 ± 0.9	93.05 ± 1.24*	31.52 ± 0.3
RNase 3	80.07 ± 0.90	34.62 ± 0.08	104.93 ± 2.80	35.55 ± 0.4
RNase 3‐H15A	67.27 ± 1.13*	29.08 ± 1.0	61.32 ± 0.63**	20.77 ± 0.2
RNase 3‐W35A	28.42 ± 0.42**	12.28 ± 0.7	24.84 ± 0.25**	8.46 ± 0.6

Maximum membrane depolarization and permeabilization activities were determined at 1 *μ*mol/L final protein concentration at final incubation time using the DiSC_3_(5) probe and *Sytox* ^*®*^ *Green*, respectively, as described in Experimental Procedures. All values, given as mean ± SD, are averaged from three replicates of two independent experiments.

^1^Arbitrary fluorescence unit (AU) values are indicated for maximum membrane depolarization and permeabilization.

^2^The calculated percentages refer to the maximum values achieved at final incubation time, referred to the positive control (10% of Triton X‐100).

The *P* value were calculated using as reference each wild‐type activity (*corresponds to *P* < 0.05 and ** to *P* < 0.009).

### A dual mode for RNases antifungal activity

The mechanism of action against *C. albicans* was then analyzed by ablating either the enzymatic activity or cell binding ability of the proteins. To assess the potential contribution of the catalytic activity of the RNases on their antimicrobial action, we prepared mutants of both RNases with defective active sites. The active site mutants were designed by making substitutions at His 15 within the active site catalytic triad (Boix et al. 1999; Huang et al. 2007), where His 15 is the counterpart of His 12 in RNase A (Fig. 1) (Cuchillo et al. 2011). The histidine to alanine substitution almost abolished all of the protein enzymatic activity for both RNases (Table 4) as previously reported by Raines and coworkers for the RNase A‐H12A mutant counterpart (Park et al. 2001). Additionally, the overall three‐dimensional structure and the active site architecture of the mutant protein were maintained, as confirmed by solving the RNase 3‐H15A mutant X‐ray crystal structure (PDB ID: 4OWZ) (Fig. 1 and Table S1). The functional characterization of both active site mutants confirmed that these mutant proteins conserved their membrane lytic activity, showing an equivalent leakage activity on lipid vesicles (Table 4). Furthermore, the potential contribution of the H15 residue on the proteins’ affinity for the cell membrane was discarded, being a residue poorly exposed to the protein surface (solvent accessible surface area, SASA, for His 15 ~ 14 Å; 4A2Y.pdb (Boix et al. 2012a)).

**Figure 3:**
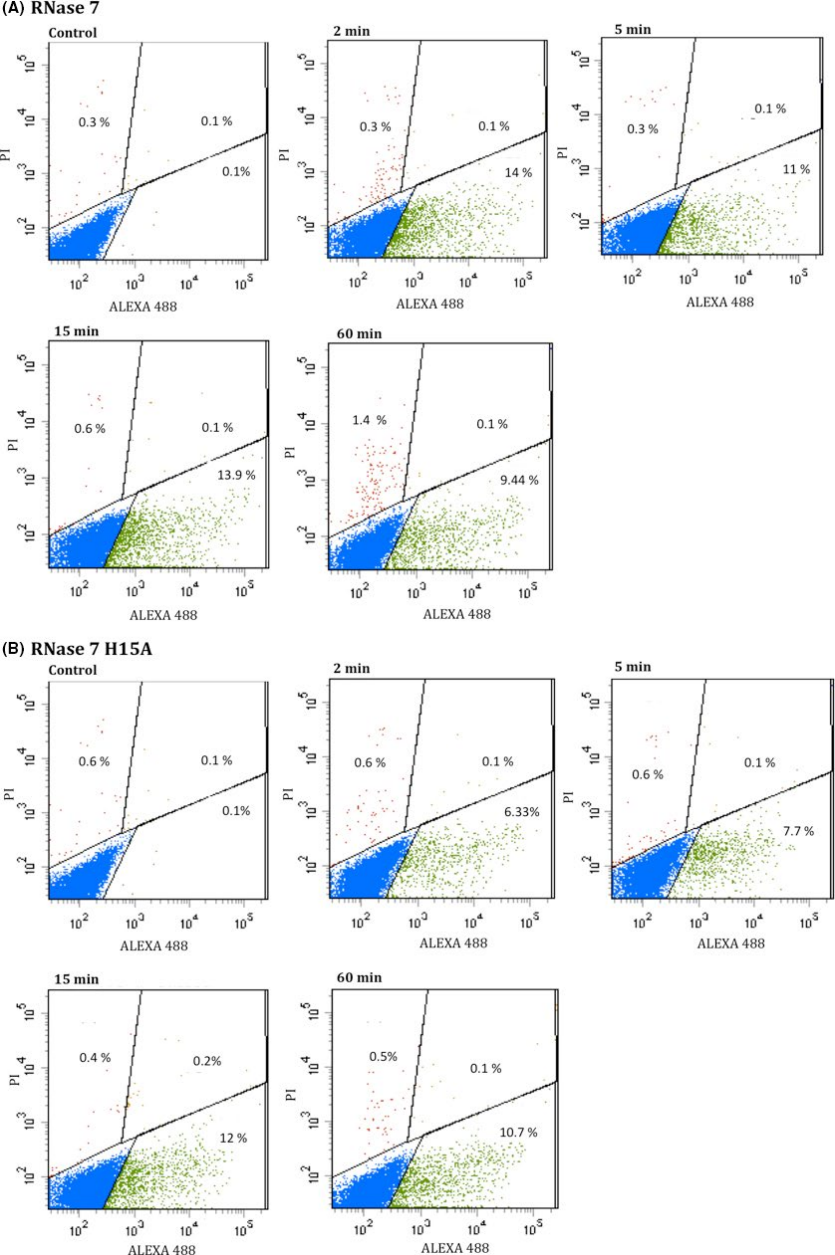
Analysis of *Candida albicans* cell culture (1 × 10^6^ cells/mL) incubated with 1 *μ*mol/L of protein by FACS. Cells were gated by Forward scatter (FSC)/Side scatter (SSC). Additionally, the incubation mixture was treated with PI to identify the dead cell population. After addition of RNase 7 (A) and RNase 7‐H15A (B) the samples were analyzed using a FACSCalibur cytometer at 2, 5, 15 and 60 min. Dot plot diagrams of Protein Alexa Fluor 488/PI show cell population divided in: free live cells (blue), cells with uptake protein (green), free dead cells (red), and dead cells with protein uptake (orange). Control corresponds to untreated cells.

Following, we compared the recombinant variants with ablated active sites with the wild‐type proteins in yeast cell cultures (Table 1). The protein activities were evaluated both by comparing the CFU count and assessing the cell viability by monitoring the ATP levels (Table 1). Differences between the cytotoxicities of the wild‐type and active site mutants were observed at sublethal concentrations and final incubation times. A kinetic time course of yeast cell viability indicated that the catalytically defective proteins showed a delay in their t_50_ values together with a lower rate of cell death at the final incubation time (Table 3). On the other hand, the active site mutations did not interfere with the protein membrane lysis activity on synthetic lipid vesicles (Table 4). Notably, depolarization time course profiles revealed no major differences between wild‐type and active site mutant proteins in comparison with the membrane binding defective mutant (Tables 2 and 3). Additionally, there was no difference in the protein binding to the yeast cells for the H15A variant, as quantified by a fluorescence assisted cell sorting (FACS) assay (Fig. 3). On the other hand, by combining FACS analysis and propidium iodide (PI) staining, we also estimated the live and dead cell subpopulations, confirming that the membranes of the cells were not significantly compromised at the assayed conditions (Figs. 3 and S4).

**Table 3:** Comparison of calculated time to achieve 50% activity (t_50_) for membrane depolarization, membrane permeabilization, and cell survival

Protein	Membr. depolarization t_50_ (sec)	Membr. permeabilization t_50_ (sec)	Cell survival t_50_ (sec)	Cell survival (%)
RNase 7	261.23	595.53	1397	38.2
RNase 7‐H15A	288.53	698.15*	1763*	54.86**
RNase 3	251.36	490.84	2354	56.87
RNase 3‐H15A	356.75*	975.5**	2965*	67.89**

All assays were carried out at 1 *μ*mol/L final protein concentration. Depolarization was assayed using DISC3(5) dye, cell leakage by the *Sytox Green* assay and survival percentage at final incubation time (120 min) was evaluated using the Live/dead® kit. T‐student was applied for comparison of numerical variables using as reference each activity corresponding to wild‐type protein, where * corresponds to *P* < 0.05 and ** to *P* < 0.009.

**Table 4:** Relative enzymatic activity was determined by the spectrophotometric method using (Cp)_4_C>p substrate as described in the Experimental Procedures section

Protein	RNase activity (%)	LUV leakage ED_50_ (*μ*mol/L)
RNase 7	100	1.14 ± 0.03
RNase 7‐H15A	9	1.24 ± 0.09
RNase 3	100	1.33 ± 0.71
RNase 3‐H15A	0	1.44 ± 0.14

Leakage of large unilamellar vesicles (LUV) is expressed as 50% effective dose (ED_50_), given as mean ± SD, averaged from three replicates of two independent experiments.

**Figure 4:**
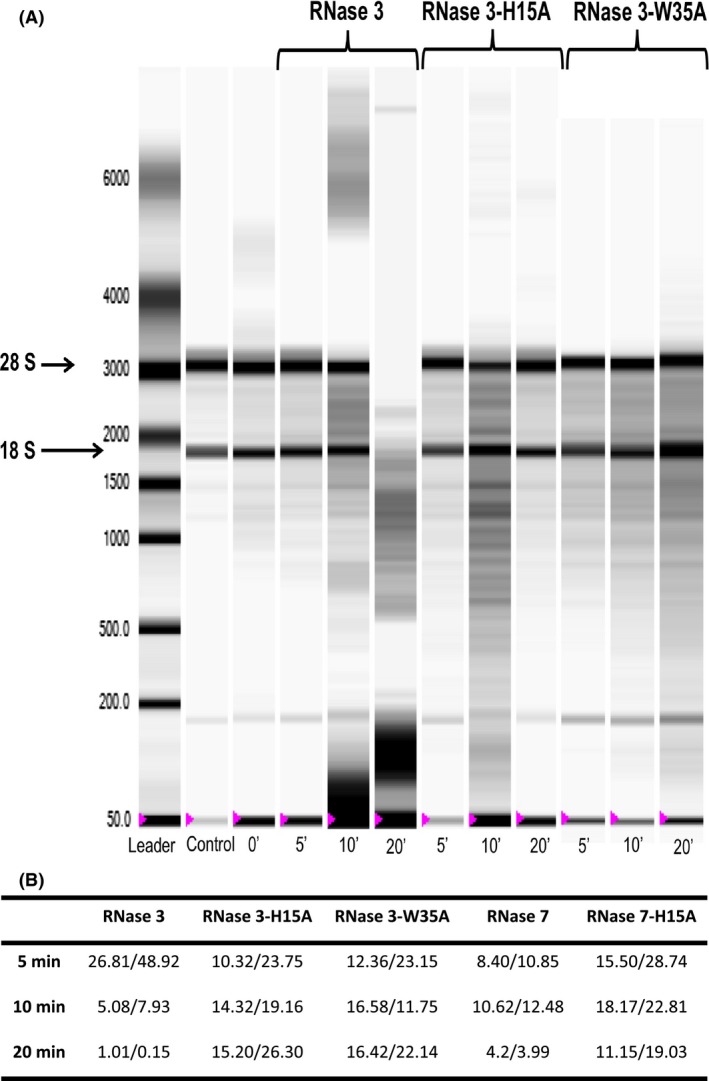
Effect of RNases on *Candida albicans* cellular RNA. 1 mL of yeast cell suspension (~ 1 × 10^7^ cells/mL) was treated with 3 *μ*mol/L of each protein and incubated at different time intervals. Following, total RNA was extracted as described in Experimental Procedures. (A) Samples were analyzed by an *Experion* automated electrophoresis system and RNA was visualized with the *Experion* software. Left lane contains molecular mass markers, where reference base pairs are indicated. Control lane corresponds to cellular RNA from untreated cells. The RNA extraction was made at different time intervals up to 20 min. (B) Peak area corresponding to 18/28s subunits of rRNA of treated cells with wild‐type and mutant RNases are shown for each incubation time.

Lastly, the potential effect of the RNases on the yeast intracellular RNA was assessed. Total cellular RNA was extracted from treated cultures and analyzed by capillary electrophoresis (Fig. 4A). To estimate the relative activity on cellular RNA, the corresponding time course of the decrease in rRNA subunits was evaluated by densitometry as a function of time (Fig. 4B). The results confirmed that there was a drastic reduction in the cellular RNA degradation rate for the active site mutants. All assays were carried out at sublethal conditions, as confirmed by a simultaneous time course monitoring of the cell culture population by optical density at 600 nm and CFU counting. Moreover, no significant reduction in the cell viability at the assayed conditions was observed by the quantification of the ATP levels during the first 15 min (Fig. S5). We even observed a slight increase in the ATP concentration at the very beginning of the incubation time, which might be attributed to a blockage of the cell protein synthesis machinery, probably induced by the RNase binding to cellular nucleic acids.

Complementarily, we assayed an RNase 3 mutant version (W35A), previously reported as defective in its protein‐membrane interaction without affecting its RNase activity (Carreras et al. 2003; Nikolovski et al. 2006; Torrent et al. 2007). The present results confirm the key role of the surface exposed Trp (Fig. 1B) in the toxicity of the protein to yeast cells. The W35A mutant displays a two to threefold reduction in its fungicidal (Table 1) and membrane destabilizing activities (Table 2). Mostly, the abilities of RNase 3 to cause membrane depolarization and disruption were severely impaired (Table 2). Indeed, by confocal microscopy, we visualized how the labeled W35A mutant does not associate to the yeast cell surface and is not internalized (Figs. 2 and S2). Furthermore, there was no significant rate of intracellular RNA cleavage, which corroborates that the protein has a defective internalization mechanism (Fig. 4).

## Discussion

There is an urgent need to develop alternative antibiotics. Exploring the mechanisms of action of our own self‐defense machinery is a promising strategy toward the design of new drugs. Human antimicrobial RNases, which are secreted upon infection and display a variety of cytotoxic activities, provide a suitable working model. In particular, several members of the vertebrate RNase A family were previously reported to display toxicity against fungal pathogens, such as RNase 5 (Hooper et al. 2003), RNase 7 (Harder and Schroder 2002; Huang et al. 2007) or RNase 8 (Rudolph et al. 2006).

In this work, we have selected the two most studied human antimicrobial RNases, the eosinophil RNase 3 and the skin‐derived RNase 7, which are upregulated upon infection (Glaser et al. 2009; Mohammed et al. 2011; Boix et al. 2012b; Becknell et al. 2015) and are directly implicated in the host defense against fungal infections (Rothenberg and Hogan 2006; Rosenberg et al. 2013). *Candida albicans* was chosen here because it is a simple eukaryotic pathogen that provides a suitable model to analyze the distinct protein targets at the cellular level. High fungicidal activities were achieved for both RNases, with MFCs on the low micromolar scale (Table 1). Together with the analysis of the RNases’ membrane damage, we have followed their cell internalization and enzymatic action. The present results highlight that the RNases have a dual mode of action. Indeed, antimicrobial RNases should be regarded as multifunctional proteins, combining an enzymatic activity and a mechanical action at the membrane level, together with other described immunomodulatory activities (Boix and Nogués 2007; Gupta et al. 2012). Similar examples of multitargeted antimicrobial proteins are available in the literature, combining intracellular targets with a variety of immunomodulating properties (Hancock and Sahl 2006; Peschel and Sahl 2006; Nicolas 2009; Haney and Hancock 2013). Unfortunately, the methodological limitations and the disparity of experimental conditions have mostly delayed the understanding of the mechanism of AMPs (Nicolas 2009; Spindler et al. 2011; Stalmans et al. 2013). It is noteworthy that many proposed roles of AMPs, such as immunomodulation or intracellular targeting, are only observed when working at sublethal assay conditions (Holm et al. 2005; Haney and Hancock 2013).

In this context, the present results once again highlight the key influence of the selection of assay conditions. Our results indicated that the labeled protein readily associated to the yeast cells at short incubation time and was subsequently internalized (Figs. 2 and S2). By tracking the cell population using a cell sorting assay combined with staining of membrane compromised cells, we confirmed that in the assayed conditions, there was no significant cell death (Figs. 3 and S4). The timing of events illustrated how cell membrane damage is only achieved at longer incubation times or higher protein concentrations. Consequently, previous results obtained at higher protein concentrations attributed RNase 3 cytotoxicity merely to cell lysis action (Carreras et al. 2003; Torrent et al. 2007; Singh and Batra 2011; Torrent et al., 2010b). Additionally, we cannot discard other complementary processes, such as the RNases’ binding to nucleic acids and blockage of the cell protein translation machinery. Indeed, a previous report on the bactericidal activity of RNase 7 already suggested its putative interaction with cellular nucleic acids (Lin et al. 2010), as reported for other cationic AMPs (Brogden 2005; Nicolas 2009).

Contribution of the RNase catalytic activity to the cytotoxic properties within the RNase A superfamily members remains a matter of controversy. Although we can find some reports on the RNase antimicrobial activity inhibition by either diethyl pyrocarbonate (DEPC) treatment or by the proteinaceous RNase inhibitor (RI) (Abtin et al. 2009), the experimental results should be interpreted with caution. Indeed, the horseshoe‐like shaped inhibitor structure can engulf the RNase inside its internal cavity, covering the cationic residues involved in the protein antimicrobial activity, as proposed for the RI interference of RNase 7 binding to LPS (Spencer et al. 2014). Similarly, treatment with DEPC would modify not only the catalytic His but other His residues exposed at the protein surface.

In any case, our results confirmed that not only are RNase 3 and RNase 7 internalized in yeast cells (Fig. 2), they also contribute by their enzymatic activities to the cell killing process Both RNase H15A variants showed impaired catalytic activity but retained their destabilization action on lipid bilayers (Table 4). Besides, both active site mutants retained their cell binding and internalization abilities (Figs. 2 and 3). On the contrary, the reduction in their fungicidal activity was significant (Table 1) and the cellular RNA degradation rates for the active site mutants were drastically reduced (Fig. 4).

In addition, the RNase 3‐W35A defective mutant was used to probe the contribution of the association of the protein to the cell surface. Residue W35 lies at a protein patch involved in interactions with lipid bilayers and extracellular matrix components (García‐Mayoral et al. 2010; Torrent et al. 2011a; Lien et al. 2013), proposed to serve as a vehicle for drug delivery (Fang et al. 2013). In particular, the W35 residue was identified to participate in the binding of LPS and glycosaminoglycans (Fan et al. 2008; García‐Mayoral et al. 2013; Pulido et al. 2013a). We can hypothesize that equivalent interactions may also contribute to the proteins’ association with the *Candida* cell wall, which is predominantly composed of glucan components. Indeed, common binding motifs are found for beta‐glucan pattern recognition proteins and other carbohydrate‐binding proteins, such as LPS and heparan sulfate. In particular, a shared binding motif for LPS and 1,3‐beta‐glucans is involved in invertebrate innate immunity (Iwanaga and Lee 2005).

Our studies on *Candida* also highlighted the requirement of the Trp residue for membrane damage, protein internalization, and cellular RNA degradation (Figs. 2, 4 and S2; Table 2). Interestingly, cell internalization is easily achieved by other members of the vertebrate RNase A family (Haigis and Raines 2003; Benito et al. 2008; Chao and Raines 2011; Sundlass et al. 2013). As an example, the cellular trafficking and ribonucleolytic activities of human RNase 5 are essential for its angiogenic action (Thiyagarajan et al. 2012). The antimicrobial RNases might also be regarded as “cell penetrating proteins.” Indeed, the intracellular routing of secretory RNases could promote their physiological role in innate immunity; a process that can be regulated by the presence of the ribonuclease inhibitor (RI), which is ubiquitous in the cytosol of mammalian cells (Lomax et al. 2014). Interestingly, very recent results on the down regulation of RI during uropathogen infections suggests a direct mechanism that facilitates the antimicrobial activity of RNases when required (Spencer et al. 2014).

Understanding the determinants that can assist protein translocation without inducing any cell damage is desired for the design of alternative targeted drugs. Although many cationic and amphipathic AMPs are classified as cationic penetrating peptides (CPPs) (Brogden 2005; Nicolas 2009; Stalmans et al. 2013), most CPP behavior has only been tested against mammalian cells (Last et al. 2013), but few peptides have been described against putative eukaryote pathogens (Do et al. 2014). Thus, the study of CPPs in yeast is an emerging field that offers promising biotechnological applications (Nekhotiaeva et al. 2004; Holm et al. 2005; Mochon and Liu 2008; Marchione et al. 2014).

In conclusion, the observed antimicrobial effective doses for both RNases were found to be in the low micromolar range, which is promising in the design of antifungal agents. Hence, the human RNases secreted from blood and epithelial cells, which combine membrane lytic and enzymatic RNase activities, could work as first line safeguard sentinels. In a wider context, the vertebrate secreted RNases could contribute to the protection of a variety of body fluids, from seminal to placental fluids, tears, or even milk (D'Alessio et al. 1991; Leonardi et al. 1995; Harris et al. 2010). Indeed, a nonspecific RNA degradation activity would represent one of the quickest and most effective ways of targeting pathogen viability. We can speculate that secreted RNases may exert a direct host defense role by the removal of pathogenic RNA, both within infected cells and resident at the extracellular matrix (Gupta et al. 2012). Therefore, as innate immunity effectors, human RNases are proving to be an ideal system for the design of nonantigenic nanodelivery tools to fight invading pathogens. AMPs offer the opportunity of finding new antifungal agents, as few effective antifungal peptides are currently available (Swidergall and Ernst 2014; Tsai et al. 2014). Thus, the study of human antimicrobial RNases provide a promising model for the design of new applied therapies against fungal infections.

## Conflict of Interest

All authors declare no conflict of interests.

## Supporting information


**Table S1**. Data collection, processing and structure refinement statistics of RNase 3‐H15A crystal structure solving.
**Table S2**. Effect of NaCl and Ca^2+^ addition on the antifungal activity of RNase 3 and RNase 7.
**Figure S1**. Binding of RNase 3 and RNase 7 to *Candida* cells.
**Figure S2**. Effects of RNase 3, RNase 3‐H15A and RNase 3‐W35A on C*. albicans* visualized by confocal microscopy.
**Figure S3.** Kinetic profile of *C. albicans* cell survival incubated with RNase 3 and RNase 7.
**Figure S4**. Analysis of C. *albicans* cell culture incubated with wild‐type and mutant RNases by FACS.
**Figure S5**. Kinetic profile of cellular ATP levels of *C. albicans* incubated with RNase 3 and RNase 3‐H15A.
