# Supplementary material for: Exploring the mechanisms of action of human secretory RNase 3 and RNase 7 against Candida albicans
Source: Microbiologyopen. 2016 Jun 8;5(5):830–45. doi: 10.1002/mbo3.373 (PMC5061719; doi:10.1002/mbo3.373)
Supplement: Supplementary file 1 — Table S1. Data collection, processing and structure refinement statistics of RNase 3‐H15A crystal structure solving. Table S2. Effect of NaCl and Ca2+ addition on the antifungal activity of RNase 3 and RNase 7. Figure S1. Binding of RNase 3 and RNase 7 to Candida cells. Figure S2. Effects of RNase 3, RNase 3‐H15A and RNase 3‐W35A on C. albicans visualized by confocal microscopy. Figure S3. Kinetic profile of C. albicans cell survival incubated with RNase 3 and RNase 7. Figure S4. Analysis of C. albicans cell culture incubated with wild‐type and mutant RNases by FACS. Figure S5. Kinetic profile of cellular ATP levels of C. albicans incubated with RNase 3 and RNase 3‐H15A. [file MBO3-5-830-s001.docx]

**SUPPORTING INFORMATION:**

**Exploring the mechanisms of action of human secretory RNase 3 and RNase 7 against *Candida albicans***

**Vivian A. Salazar^1^; Javier Arranz-Trullen^1^; Susanna Navarro^1,2^; Jose A. Blanco^1^;Daniel Sánchez^1^; Mohammed Moussaoui^1^; and Ester Boix^1^**

**^1^** From the Department of Biochemistry and Molecular Biology, Faculty of Biosciences, Universitat Autònoma de Barcelona, E-08193 Cerdanyola del Vallès, Spain and ^2^Institut de Biotecnologia i Biomedicina, Universitat Autònoma de Barcelona, E-08193

Cerdanyola del Vallès, Spain. E –mail: [Ester.Boix@uab.cat](mailto:Ester.Boix@uab.cat)

**List of supplementary material**:

**Table S1**. Data collection, processing and structure refinement statistics of RNase 3-H15A crystal structure solving.

**Table S2**: Effect of NaCl and Ca^2+^ addition on the antifungal activity of RNase 3 and RNase 7*.*

**Figure S1. Binding of RNase 3 and RNase 7 to *Candida* cells.**

**Figure S2**: Effects of RNase 3, RNase 3-H15A and RNase 3-W35A on C*. albicans* visualized by confocal microscopy.

**Figure S3:** Kinetic profile of *C. albicans* cell survival incubated with RNase 3 and RNase 7.

**Figure S4**: Analysis of C. *albicans* cell culture incubated with wild-type and mutant RNases by FACS.

**Figure S5**. Kinetic profile of cellular ATP levels of *C. albicans* incubated with RNase 3 and RNase 3-H15A. **Table S1**. Data collection, processing and structure refinement statistics of RNase 3-H15A crystal structure solving.

|  | **RNase 3-H15A**  **(PDB ID: 4OWZ)** |
| --- | --- |
| *Data collection* |  |
| Space group | P4_3_22 |
| Unit cell |  |
| *a, b, c* (Å) | 62.54 62.54 175.23 |
| α, β, γ (°) | 90.0 90.0 90.0 |
| Resolution (Å) | 1.47 |
| Number of reflections (measured/unique) | 775132/48793 |
| R_merge_ ^a, b^(%) | 4.2 (44.7) |
| *I/σ_Ι_* ^b^ | 34.9 (6.8) |
| Completeness for range (%) ^b^ | 100.0 (100.0) |
| Wilson B factor (Å^2^) ^b^ | 21.6 |
| Matthews coefficient (Å^3^/Da) ^b^ | 2.20 |
| Solvent content (%) | 43.59 |
| *Refinement parameters* |  |
| Resolution range (Å) | 62.54 – 1.47 |
| R_cryst_ ^c^ / R_free_ ^d^ (%) | 20.02/22.25 |
| Bond lengths (Å) | 0.007 |
| Bond angles (deg) | 1.115 |
| Number of protein atoms | 2335 |
| Number of water molecules | 413 |
| B-factors (Å^2^) |  |
| Protein atoms |  |
| - All | 23.05/26.22 |
| - Main chain | 20.63/23.39 |
| - Side chain | 25.03/28.70 |
| Anion atoms | 27.89 |
| Water molecules | 39.15 |

*^a^ R_merge_=* Σ*_hkl_* Σ*_j-1_* to *N*|*I_hkl_-I_hkl_(j)*|/ Σ*_hkl_* Σ*_j-1_* to *N* *I_hkl_(j), where N is the redundancy of the data.*

*^b^ Outermost shell is 1.47-1.62*

*^c^ R_crystal_*= Σ*_h_*|*F_o_-F_c_*|/Σ*_h_F_o_, where F_o_ and F_c_ are the observed and calculated structure factor amplitudes of reflection* h*, respectively.*

*^d^ R_free_ is equal to R_cryst_ for a randomly selected 5% subset of reflections not used in the refinement.*

**Table S2**: Effect of NaCl and Ca^2+^ addition on the antifungal activity of RNase 3 and RNase 7*.* The minimal fungicidal concentration (MFC_100_) was determined on *Candida albicans* cultures diluted in Low ionic strength Phosphate Buffer (Low-PB: 10 mM sodium phosphate, pH 7.5); High ionic strength Phosphate Buffer (High-PB: 10 mM sodium phosphate, NaCl 0.15M, pH 7.5) and Sabouraud Broth (SB) by CFU counting on plated Petri dishes as described in the methodology.

| Protein | MFC_100_ (μM) | | | | |
| --- | --- | --- | --- | --- | --- |
|  | Low-PB | High-PB | SB | SB + 150 mM NaCl | SB + 1 mM Ca^2+^ |
| RNase 7 | 2.5-5 | 2.5 | 2.5-5 | 5-10 | 20 |
| RNase 3 | 2.5-5 | 2.5 | 2.5 | 10 | >20 |

**SUPPLEMENTAL FIGURES**

**
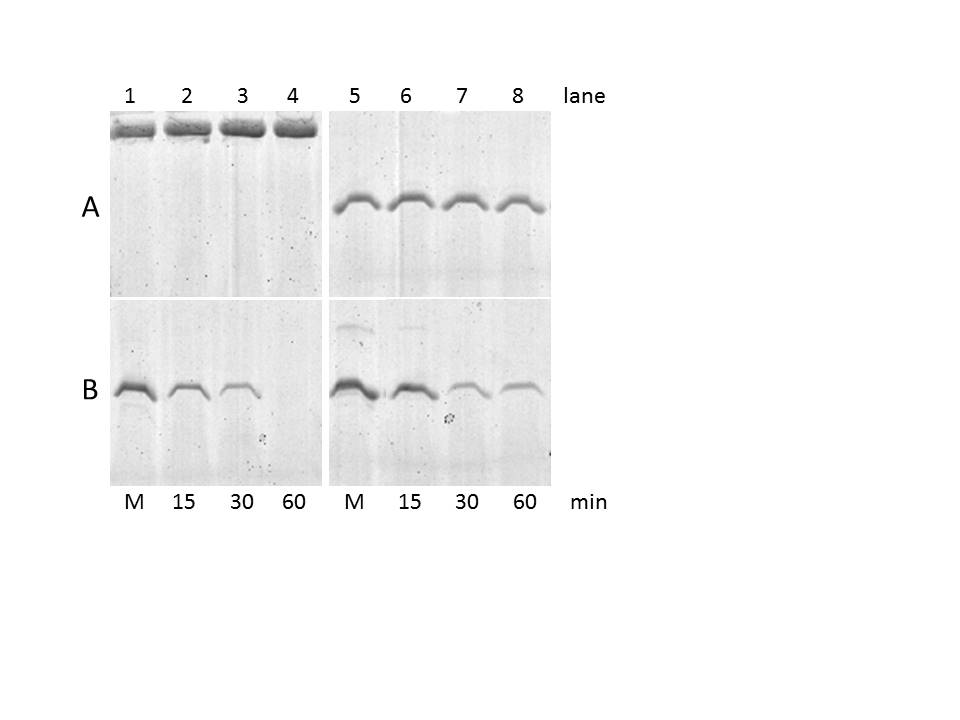
**

**Figure S1. Binding of RNase 3 and RNase 7 to *Candida* cells.** Lanes 1A-4A correspond to bovine serum albumin (BSA). Lanes 5A-8A correspond to RNase A. Lanes 1B-4B correspond to RNase 3. Lanes 5B-8B correspond to RNase 7. Lanes 1A, 5A, 1B and 5B correspond to reference proteins.

**
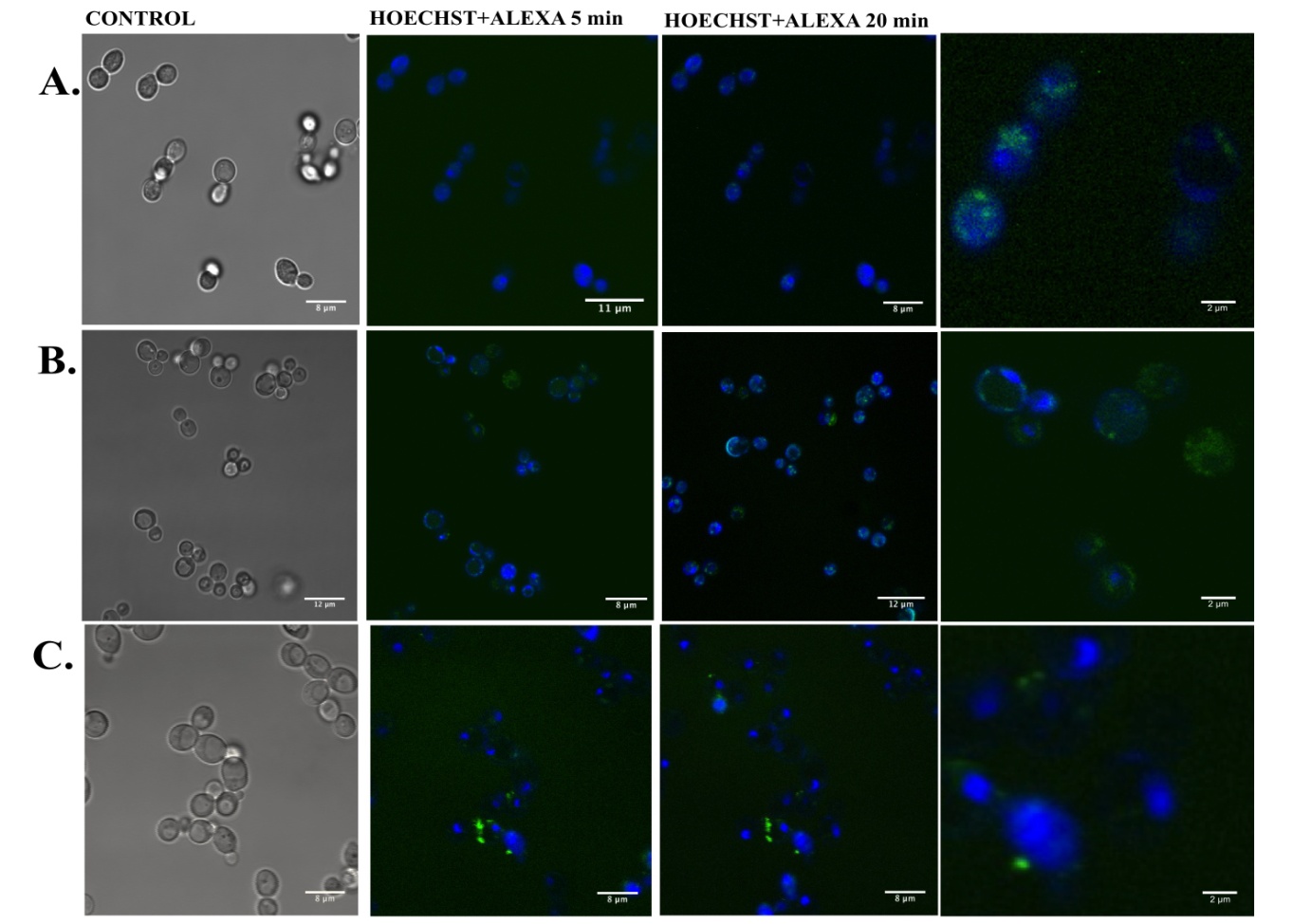
**

**Figure S2**: Effects of RNase 3, RNase 3-H15A and RNase 3-W35A (A-C) on C*. albicans* in mid-exponential growth phase (~3 x10^6^ cell/mL) visualized by confocal microscopy. The yeast morphology is showing in the left-hand side. The second and third panels show the merged Hoechst and Alexa Fluor 488– labeled protein after 5 and 20 min of incubation respectively. Last panel corresponds to a magnified window of the 20 min incubation condition. The protein final concentration was 3 μM. *ImageJ* software was used for analysis. The magnification scale is indicated at the bottom of each micrograph. Images were taken using a Leica TCS SP5 AOBS microscope.

**
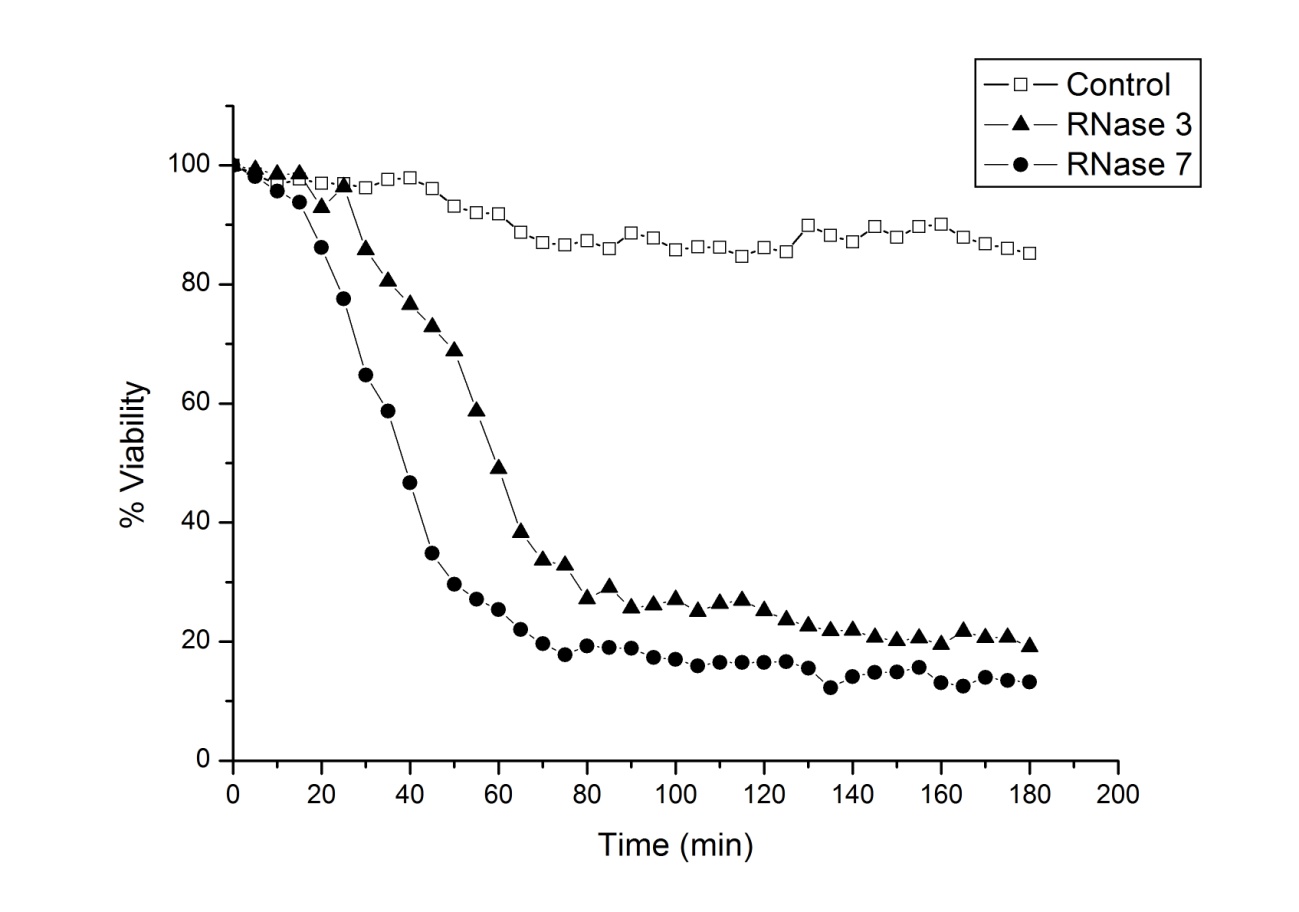
**

**Figure S3:** Kinetic profile of calculated *Candida albicans* cell survival after incubation of yeast cell culture (~ 2 x 10^6^ cells/mL) with 1 μM of RNase 3 and RNase 7 at 37ºC. Viability percentage was evaluated using the Live/Dead^®^ kit, where live and dead cells are stained with SYTO^®^9 and propidium iodide (PI) dyes respectively.


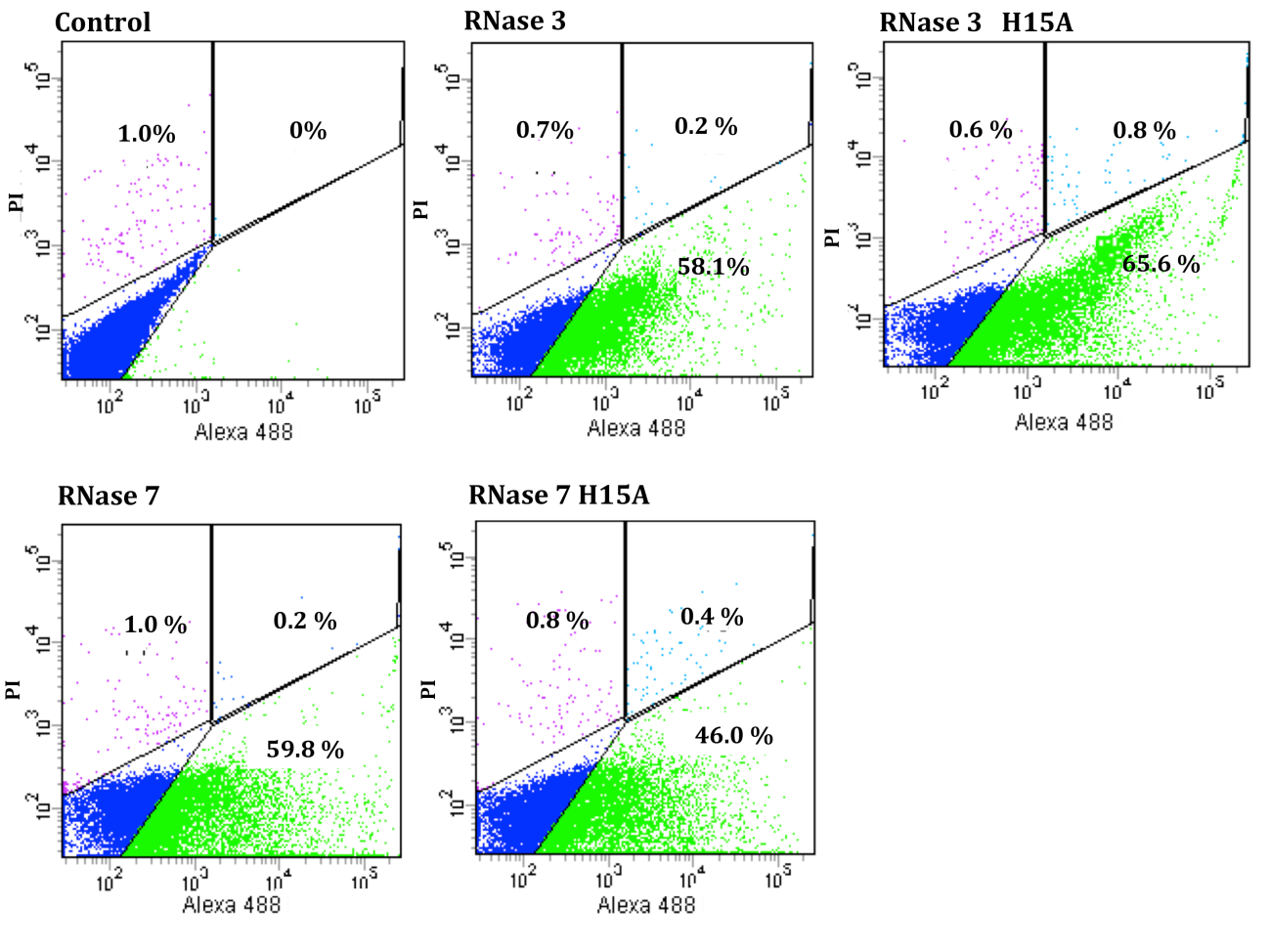


**Figure S4**: Analysis of C. *albicans* cell culture incubated with proteins by FACS. A total of 25000 *Candida* cells were gated by Forward scatter (FSC) / Side scatter (SSC). *C.* *albicans* cell cultures (1x10^6^ cells/mL) were incubated with 3.5 μM of RNase 3, RNase 7 and mutants during 45 min; the samples were analyzed using a FACSCalibur cytometer. Dot plot diagrams of Protein-Alexa Fluor 488/ PI show yeast population divided in four quadrants corresponding to: *Candida cells* (blue color), cells with colocalized protein (green color), dead cells (purple color) and dead cells with colocalized protein (light blue color). Control corresponds to untreated cells.


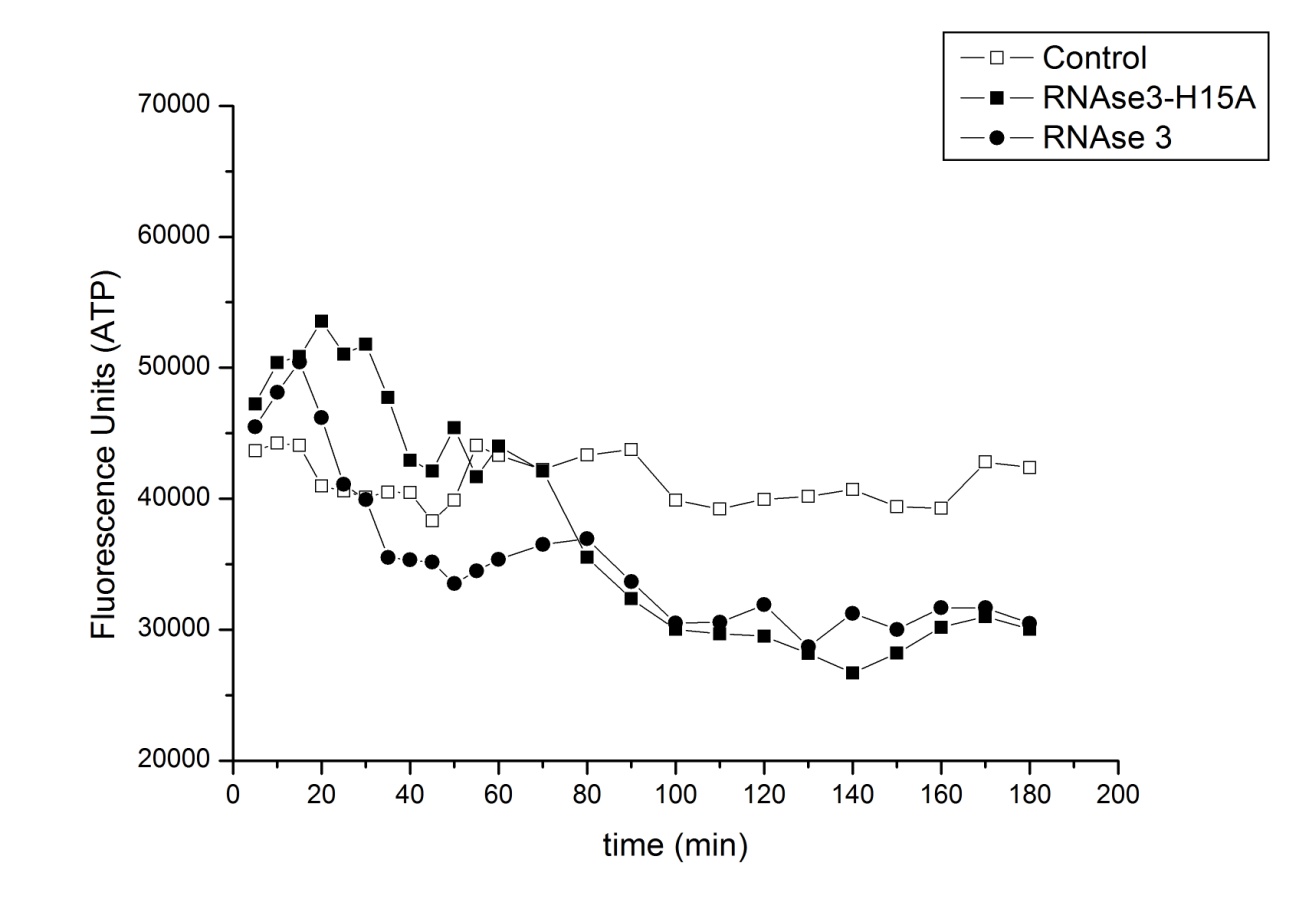


**Figure S5**. Kinetic profile of cellular ATP levels assessed by using the *Bac-Titer Glo*^TM^ Assay. Yeast cell cultures grown up to ~3 x10^6^ cells/mL were incubated with 1 μM of RNase 3 and RNase 3-H15A at 37ºC.
